# Role of Palmitoylation of Postsynaptic Proteins in Promoting Synaptic Plasticity

**DOI:** 10.3389/fnmol.2019.00008

**Published:** 2019-01-31

**Authors:** Lucas Matt, Karam Kim, Dhrubajyoti Chowdhury, Johannes W. Hell

**Affiliations:** ^1^Department of Pharmacology, Toxicology and Clinical Pharmacy, Institute of Pharmacy, University of Tübingen, Tübingen, Germany; ^2^Department of Pharmacology, University of California, Davis, Davis, CA, United States

**Keywords:** AMPAR, NMDAR, LTP, LTD, homeostatic plasticity, PSD-95

## Abstract

Many postsynaptic proteins undergo palmitoylation, the reversible attachment of the fatty acid palmitate to cysteine residues, which influences trafficking, localization, and protein interaction dynamics. Both palmitoylation by palmitoyl acyl transferases (PAT) and depalmitoylation by palmitoyl-protein thioesterases (PPT) is regulated in an activity-dependent, localized fashion. Recently, palmitoylation has received attention for its pivotal contribution to various forms of synaptic plasticity, the dynamic modulation of synaptic strength in response to neuronal activity. For instance, palmitoylation and depalmitoylation of the central postsynaptic scaffold protein postsynaptic density-95 (PSD-95) is important for synaptic plasticity. Here, we provide a comprehensive review of studies linking palmitoylation of postsynaptic proteins to synaptic plasticity.

## Introduction

Glutamate is the neurotransmitter used by the vast majority of synapses in the brain for fast excitatory neurotransmission (Micheva et al., 2010). It activates postsynaptic AMPA-type glutamate receptors (AMPAR) that are located precisely juxtaposed to presynaptic release sites. Specific patterns of synaptic activity lead to activation of NMDA-type glutamate receptors (NMDAR), causing postsynaptic Ca^2+^ influx. Large increases in intracellular Ca^2+^ strengthen the synapse by increasing the postsynaptic number of AMPAR, a process called long-term potentiation (LTP), while smaller, more prolonged ones reduce postsynaptic AMPAR numbers causing long-term depression (LTD; Huganir and Nicoll, 2013; Diering and Huganir, 2018). These plastic changes in the efficiency of glutamatergic synapses are thought to represent the physiological basis of learning and memory (Lüscher and Malenka, 2012; Takeuchi et al., 2014). Both LTP and LTD have been most intensively studied in the hippocampus, a brain region involved in spatial and declarative learning and memory.

**Figure 1:**
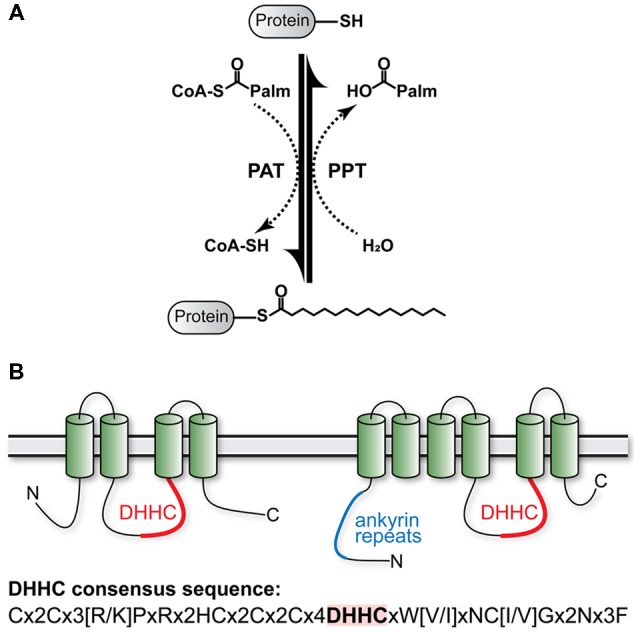
Protein Palmitoylation. **(A)** The thioester bond of palmitic acid (palm), which is covalently linked to coenzyme A (CoA), is transferred to cysteine by specialized palmitoyl acyl-transferases (PAT). Selective palmityol protein-thioesterases (PPT) catalyze hydrolytic cleavage of palmitic acid from cysteine moieties. **(B)** Membrane topology of ZDHHC proteins. ZDHHCs form four (left) or six (right) transmembrane domains (TMDs) with N- and C-termini in the cytoplasm. The conserved DHHC domain is indicated in red, ankyrin repeats are indicated in blue. The consensus sequence of the DHHC domain is given at the bottom (Nadolski and Linder, 2007).

The postsynaptic density (PSD), originally identified as an electron-dense area by electron microscopy, is a protein-rich region located beneath the postsynaptic membrane of a neuron. Besides the aforementioned glutamate receptors, it contains several hundred different proteins essential for synaptic functions including scaffolding proteins, neurotransmitter receptors, cell-adhesion molecules, and signaling molecules such as kinases and phosphatases. Through the action of these molecules, the PSD is a central conduit for transmission of neuronal signals. It undergoes changes in its composition and morphology depending on the neural activity in order to process the information (Kennedy, 2000; Kim and Sheng, 2009). Posttranscriptional modifications of AMPAR, NMDAR, and a number of other abundant proteins of the PSD are either direct mechanisms of synaptic plasticity or control the ability of synapses to undergo plasticity. These include phosphorylation (Woolfrey and Dell’Acqua, 2015), ubiquitination (Mabb and Ehlers, 2010; Lin and Man, 2013), S-nitrosylation (Bradley and Steinert, 2016), neddylation (Vogl et al., 2015), and palmitoylation. Palmitoylation is the reversible covalent acylation of cysteine residues by attachment of long-chain fatty acids through thioester-bonds (Figure 1A). The majority of transferred acyls stems from the 16-C fatty acid palmitic acid. Hence, this protein modification is commonly referred to as S-palmitoylation. The addition of a hydrophobic residue serves to target proteins to cholesterol-rich sections (lipid rafts) of intracellular organelles or the plasma membrane. Besides trafficking, localization, and membrane microdomain association, addition of palmitate to cysteine residues can alter a number of protein properties like conformation, complex formation, and interplay with other post-translational modifications like phosphorylation and ubiquitination (reviewed in Blaskovic et al., 2013; and Blaskovic et al., 2014). Attachment of palmitoylate residues is catalyzed by specialized palmitoyl acyl-transferases (PAT) and depalmitoylation by specialized palmitoyl protein thioesterases (PPT).

In recent years, the contribution of protein palmitoylation to synapse development and synaptic plasticity was elucidated in more detail. The amount of effort invested in this endeavor is witnessed by a great number of review articles discussing various aspects of palmitoylation in the neuronal system (Fukata and Fukata, 2010; Thomas and Huganir, 2013; Fukata et al., 2015; Han et al., 2015; Cho and Park, 2016; Globa and Bamji, 2017; Naumenko and Ponimaskin, 2018; Zaręba-Kozioł et al., 2018). It is indeed the nervous system, in which protein palmitoylation seems to be particularly important. A recent study combining data from 15 proteomic analyses suggests that at least 10% of the known gene products are modified by palmitoylation. There is a remarkable preponderance of palmitoylation for synaptic proteins, with 41% apparently being substrates for palmitoylation (Sanders et al., 2015). It is noteworthy that there is no specific kind of protein nor any cellular function that predestines a protein for palmitoylation. The long lists of neuronal targets for palmitoylation include presynaptic proteins (Prescott et al., 2009), cytosolic proteins like protein kinases (PKAs; Montersino and Thomas, 2015), and scaffold proteins (see below) as well as integral membrane proteins like neurotransmitter receptors (Naumenko and Ponimaskin, 2018) or ion channels (Shipston, 2014).

In addition to our improved understanding of the mechanism of palmitoylation and depalmitoylation, there is also increasing knowledge about the role of palmitoylation in the pathophysiology of a variety of neurological and psychiatric diseases including Alzheimer’s and Huntington’s diseases, schizophrenia, intellectual disability and major depressive disorder (reviewed in Cho and Park, 2016; Zaręba-Kozioł et al., 2018). It is very interesting to see that several of these diseases are characterized by mutation or dysregulation of PPT (De and Sadhukhan, 2018).

Here, we review the role of palmitoylation in synaptic plasticity with focus on postsynaptic proteins for which palmitoylation is confirmed to contribute to their function in synaptic strength. We particularly emphasize PSD-95, a postsynaptic scaffolding protein with a central role in synaptic plasticity for which the regulation by palmitoylation is best characterized.

## Dynamic Palmitate Cycling and Activity

S-palmitoylation is catalyzed by a family of PAT (ZDHHC) containing a conserved DHHC (Asp-His-His-Cys) catalytic motif within a cysteine rich zinc finger-like domain (Figure 1B). ZDHHC protein structure and catalytic function are well covered in other reviews (Korycka et al., 2012; Cho and Park, 2016; Gottlieb and Linder, 2017; De and Sadhukhan, 2018). Thus, we will only provide a brief overview here. So far, 23 ZDHHC genes are known in humans. Most ZDHHCs are predicted to form four transmembrane domains (TMDs) with the exception of ZDHHC13 and ZDHHC17, which are predicted to form six TMD (Figure 1B). While the DHHC domain is highly conserved, the cytoplasmic N- and C-termini vary considerably between the different proteins. These highly diverse N- and C-termini mediate protein-protein interactions that confer at least some of the substrate specificity of ZDHHCs. There is evidence that the DHHC domain indeed binds two zinc ions as necessary structural components (Gottlieb and Linder, 2017). When these proteins were originally cloned, they were referred to as “DHHC” (Fukata et al., 2004; Ohno et al., 2006). Later, the proteins were mapped to their respective genes as “ZDHHC” by the HGNC (HUGO gene nomenclature committee) or correspondingly as “Zdhhc” by the MGI (Mouse Genome Informatics) database. The ZDHHC/Zdhhc gene names mostly correspond to the original DHHC numbers used by Fukata et al. (2004) and Ohno et al. (2006) with a few exceptions (DHHC-10 is ZDHHC11, DHHC-11 is ZDHHC23, DHHC-13 is ZDHHC24, DHHC-22 is ZDHHC13, and DHHC-23 is ZDHHC25; see Table 1). ZDHH10 is omitted, as ZDHHC11 is encoded by two independent genomic loci (ZDHHC11a and ZDHHC11b) that give rise to an identical gene product. Nowadays, authors usually denote ZDHHC palmitoyl transferases as “DHHC,” but use the numbering of the ZDHHC system. In this review article, we use the term ZDHHC and would encourage others to follow this example in the future to avoid adding confusion to the literature. The majority of ZDHHCs localizes to Golgi and ER membranes. ZDHHC2, ZDHHC5, ZDHHC11, ZDHHC20, and ZDHHC21, were described at the plasma membrane (Korycka et al., 2012; Oku et al., 2013; Cho and Park, 2016). ZDHHC2 (Noritake et al., 2009), ZDHHC5 (Thomas et al., 2012), ZDHHC8 (Mukai et al., 2004), and ZDHHC17 (Huang et al., 2004) were observed in dendritic vesicles in neurons. Interestingly, ZDHHC show broad substrate specificity which is overlapping, i.e., certain substrates are palmitoylated by more than one ZDHHC (Globa and Bamji, 2017; De and Sadhukhan, 2018). Some substrate specificity is achieved by specific interaction motifs in the intracellular N- and C-termini. Particularly interesting is the PDZ ligand in the C-termini of ZDHHC3, ZDHHC5, and ZDHHC8, which facilitates interaction with postsynaptic PDZ proteins (Gottlieb and Linder, 2017). PDZ binding of ZDHHC5 and ZDHHC8 is essential for the palmitoylation of glutamate receptor interacting protein 1b (GRIP1b; Thomas et al., 2012) and protein interacting with C-kinase 1 (PICK1; Thomas et al., 2013), respectively. Another example for a specific interaction motif is the Src homology 3 (SH3) domain of ZDHHC6, which mediates the interaction with selenoprotein K (SelK). A ZDHHC6-SelK complex is necessary for the palmitoylation of the inositol 1,4,5-triphosphate receptor (Fredericks et al., 2014). The N-terminal ankyrin repeats found in ZDHHC13 and ZDHHC17 also mediate specific interactions with a number of proteins including SNAP23, SNAP25, and huntingtin (Lemonidis et al., 2015).

**Table 1:** ZDHHC proteins in mouse and human.

Mouse gene name	Human gene name	DHHC identifier/ frequent synonyms	Mouse RefSeq ID	Human RefSeq ID	Expression in neurons (Globa and Bamji, 2017)	Subcellular localization (Korycka et al., 2012; Cho and Park, 2016; Globa and Bamji, 2017; De and Sadhukhan, 2018)	Synaptic plasticity target
Zdhhc1	ZDHHC1		NM_175160.3	NM_013304	Hippocampus (Cajigas et al., 2012; Oku et al., 2013)	Dendrites, early endosomes after overexpression (Oku et al., 2013)	
Zdhhc2	ZDHHC2	Ream	NM_178395	NM_016353	Cortex, hippocampus (Noritake et al., 2009; Woolfrey et al., 2015; Cembrowski et al., 2016)	Dendrites, translocates from shaft to spine after chronic activity blockade (Noritake et al., 2009; Woolfrey et al., 2015)	AKAP5 (Woolfrey et al., 2015), PSD-95 (Fukata et al., 2013)
Zdhhc3	ZDHHC3	GODZ	NM_026917	NM_016598	Noritake et al. (2009); Thomas et al. (2012)	Somatic Golgi (Noritake et al., 2009; Thomas et al., 2012)	GluA1 (Hayashi et al., 2005)
Zdhhc4	ZDHHC4		NM_028379	NM_018106	Levy et al. (2011)	Endoplasmic reticulum when overexpressed (Levy et al., 2011)	
Zdhhc5	ZDHHC5		NM_144887	NM_015457	Ubiquitously expressed (Thomas et al., 2012; Brigidi et al., 2015)	Dendrites, translocates from plasma membrane to endosomes (Thomas et al., 2012; Brigidi et al., 2015)	δ-catenin (Brigidi et al., 2014), GRIP1b (Thomas et al., 2012)
Zdhhc6	ZDHHC6		NM_025883	NM_022494			
Zdhhc7	ZDHHC7	SERZ1	NM_133967	NM_017740	Cortex, hippocampus, olfactory bulb (Thomas et al., 2012)	Somatic Golgi (Thomas et al., 2012)	
Zdhhc8	ZDHHC8		NM_172151.4	NM_013373	Cortex, hippocampus, olfactory bulb (Thomas et al., 2012)	Dendrites (Thomas et al., 2012)	Cdc42 (Mukai et al., 2008; Moutin et al., 2017), PICK1 (Thomas et al., 2013)
Zdhhc9	ZDHHC9		NM_172465.4	NM_016032	Ubiquitously expressed (Swarthout et al., 2005)		
Zdhhc11	ZDHHC11	DHHC-10	NM_027704.2	NM_024786			
	ZDHHC11B			NM_001351303			
Zdhhc12	ZDHHC12		NM_001037762	NM_032799	Dejanovic et al. (2014)	Somatic Golgi, in Golgi outposts when overexpressed (Dejanovic et al., 2014)	Gephyrin (Dejanovic et al., 2014)
Zdhhc13	ZDHHC13	DHHC-22, HIP14L	NM_028031	NM_019028	Hippocampus (Cajigas et al., 2012)		
Zdhhc14	ZDHHC14		NM_146073.3	NM_153746	Hippocampus (Cajigas et al., 2012)		
Zdhhc15	ZDHHC15		NM_175358	NM_144969	Hippocampus (Cajigas et al., 2012)		
Zdhhc16	ZDHHC16	APH2	NM_023740.2	NM_032327			
Zdhhc17	ZDHHC17	HIP14	NM_172554	NM_015336	Ubiquitous expression (Globa and Bamji, 2017)	Golgi (Huang et al., 2004)	
Zdhhc18	ZDHHC18		NM_001017968.2	NM_032283			
Zdhhc19	ZDHHC19		NM_199309.2	NM_144637			
Zdhhc20	ZDHHC20		NM_029492.4	NM_153251	Cortex hippocampus hypothalamus, (Cajigas et al., 2012)		
Zdhhc21	ZDHHC21		NM_026647	NM_178566	Hippocampus (Cajigas et al., 2012)		
Zdhhc22	ZDHHC22		NM_001080943	NM_174976	Thalamus, Hippocampus (Cajigas et al., 2012)		
Zdhhc23	ZDHHC23	DHHC-11	NM_001007460.1	NM_173570	Hippocampus (Cajigas et al., 2012)		
Zdhhc24	ZDHHC24	DHHC-13	NM_027476.2	NM_207340			
Zdhhc25		DHHC-23	NM_027306.2				

*Mouse and human gene names accord to the standard nomenclature by the Mouse Genome Informatics (MGI) database and the HUGO gene nomenclature committee (HGNC), respectively. DHHC refers to the nomenclatures originally used by Fukata et al. (2004) and Ohno et al. (2006). The table only lists DHHC numbers for proteins where it differs from the ZDHHC nomenclature*.

The palmitoylation state of any protein is controlled by the opposing processes of palmitoylation and depalmitoylation. Initial palmitoylation of newly synthesized substrates occurs at the Golgi apparatus where most ZDHHC PATs are localized. Such lipidation promotes tethering to membranes or sorting to particular lipid microdomains and allows trafficking of the substrates through the secretory pathway (McCormick et al., 2008). PATs are also present at several endomembrane compartments and the plasma membrane where local palmitate cycling can alter the localization, structure, and function of their target proteins.

Depalmitoylation, on the other hand, has been suggested to occur ubiquitously, including at the plasma membrane (Rocks et al., 2010). Much less is known about depalmitoylating PPT enzymes. The acyl-PPT APT1, APT2, and APT-like are located in the cytosol, while the palmitoyl-PPT PPT1 and PPT2 are expressed in lysosomes (Lin and Conibear, 2015b). Furthermore, recent work identified the ABDH family of depalmitoylating proteins (Lin and Conibear, 2015a; Yokoi et al., 2016). The role of reversible protein palmitoylation in the dynamic shuttling of palmitoylated proteins between the Golgi apparatus and the peripheral membranes has been demonstrated in non-polarized cells (Rocks et al., 2005; Chisari et al., 2007). Such regulation of protein localization and/or function by palmitoylation is expected to be especially critical in highly polarized cells such as neurons. Here, we highlight a number of studies elucidating how the subcellular locus of palmitoylation/depalmitoylation in neurons is possibly related to neuronal activity. The small Rho GTPase cell division cycle 42 (cdc42), a ubiquitous regulator of the actin cytoskeleton, moves in and out of dendritic spines upon glutamate treatment of cultured neurons in a palmitoylation-dependent manner (Kang et al., 2008). More recently, local depalmitoylation of microtubule-associated protein 6 (MAP6) within axons was found to mediate its detachment from secretory vesicles and targeting to axonal microtubules in a Ca^2+^-dependent manner although the precise regulatory mechanism remains unknown (Tortosa et al., 2017). Given the overlapping substrate specificities of the palmitoylating/depalmitoylating enzymes, activity-driven changes in palmitate turnover can arise from either targeting of the enzymes to the relevant substrate or accessibility of the thiol or thioester groups on the substrate, which may depend on conformational changes or altered interaction with other proteins. One example of such activity-dependent targeting of the enzyme is the recruitment of ZDHHC2 to the PSD where it augments palmitoylation of PSD-95 thereby enhancing its synaptic localization upon chronic activity blockade with tetrodotoxin (TTX; Noritake et al., 2009). Another study showed that enhanced postsynaptic activity during chemical LTP (cLTP) ejects the plasma membrane-localized ZDHHC5 from dendritic spines to shafts, where it palmitoylates δ-catenin on recycling endosomes (RE) promoting its forward trafficking into spines (Brigidi et al., 2015). Palmitoylation of target proteins can also be regulated indirectly by external cues through modified catalytic activity of the enzymes. For example, tyrosine phosphorylation of ZDHHC3 on its cytosolic face by stimulation of FGF receptor 1 (FGFR1) antagonized its auto-palmitoylation and interaction with and palmitoylation of neural cell adhesion molecule (NCAM; Lievens et al., 2016). However, presence of multiple palmitoylation sites independently targeted by distinctly localized enzymes makes it difficult to assess the effect of activity on native protein palmitoylation in absence of site-specific antibodies. While the GluA1 and GluA2 subunits of AMPAR are palmitoylated at the C-terminal end of the second transmembrane domain (TMD2) and on the intracellular C-terminal domain, only the C-terminal palmitoylation specifically enhances susceptibility to agonist-induced internalization (Hayashi et al., 2005), although the overexpression strategy used in this study might have caused secondary effects.

## Plasticity-Related Palmitoylation of Postsynaptic Proteins

### Palmitoylation of Neurotransmitter-Receptors and Receptor-Associated Proteins

A substantial number of receptors for a variety of neurotransmitters are palmitoylated (reviewed in Naumenko and Ponimaskin, 2018). The majority of G protein-coupled receptors contain putative palmitoylation sites (Probst et al., 1992), but palmitoylation was also established for a number of ionotropic, ligand-gated ion channels including AMPAR, NMDAR, kainate receptors (Pickering et al., 1995), the γ_2_-subunit of the γ-aminobutyric acid type A (GABA_A_) receptor (Keller et al., 2004; Rathenberg et al., 2004), the α_4_-subunit of the nicotinic acetylcholine receptor (Amici et al., 2012), and the adenosine triphosphate receptor P2X7 (Gonnord et al., 2009). So far, however, dynamic regulation of palmitoylation during the expression of synaptic plasticity was only shown for AMPAR and NMDAR.

#### AMPAR

**Figure 2:**
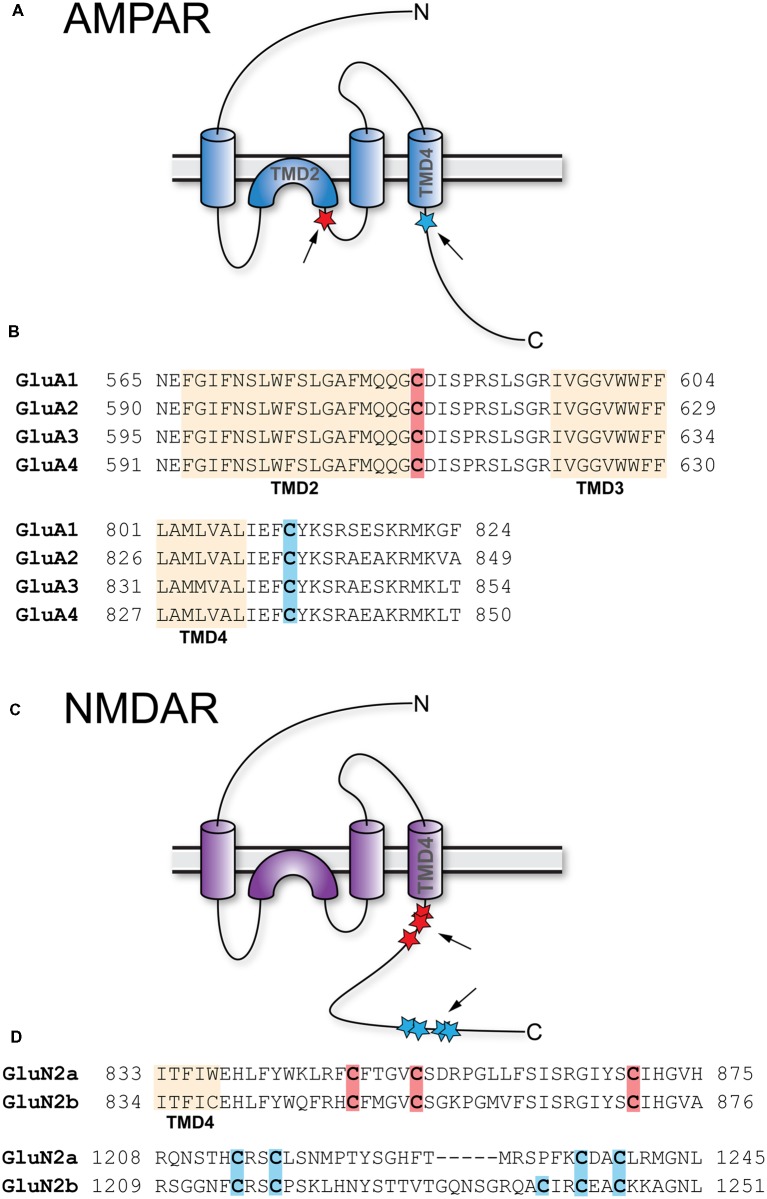
Palmitoylation of AMPA-type glutamate receptors (AMPAR) and NMDA-type glutamate receptors (NMDAR). **(A)** Topology of AMPAR subunits. **(B)** Sequence alignment of the GluA1–4 regions that harbor palmitoylation sites. **(C)** Topology of NMDAR subunits. **(D)** Sequence alignment of the GluN2A and GluN2B regions that harbor palmitoylation sites. Palmitoylation sites are indicated by red and blue stars and arrows in **(A,C)**. Orange shading in **(B,D)** indicates TMD, red and blue shading cysteines corresponding to red and blue stars in **(A,C)**.

The AMPAR is a tetramer formed by four subunits, which are homologous to each other: GluA1, GluA2, GluA3, and GluA4. In the adult, AMPAR are predominantly comprised of GluA1 and GluA2 (Traynelis et al., 2010). All four GluA subunits feature two palmitoylation sites (Figure 2A). One is located in TMD2 (GluA1 C585, GluA2 C610, GluA3 C615, GluR4 C611), the other in the C-terminal tail (GluA1 C811, GluA2 C836, GluA3 C841, GluA4 C817; Hayashi et al., 2005; Figure 2B). Overexpression of ZDHHC3 causes palmitoylation of GluA1 and GluA2 at their TMD2, which results in their retention in the Golgi apparatus (Hayashi et al., 2005). Endogenous palmitoylation of TMD2 might constitute part of a quality control step in receptor formation (Yang et al., 2009). Palmitoylation at their C-terminal site does not affect steady state expression of GluA1 and GluA2. When cysteine to serine mutation renders the C-terminal site of GluA1 and GluA2 palmitoylation-deficient, however, AMPAR are not endocytosed after glutamate or NMDA treatment (Hayashi et al., 2005). This effect is due to the fact that PKC-mediated phosphorylation of GluA1 at Ser816 and Ser818 is increased when the C-terminal site is not palmitoylated, which in turn enhances the stabilizing interaction between GluA1 and 4.1N, preventing receptor internalization (Hayashi et al., 2005; Lin et al., 2009). This palmitoylation-dependence of receptor internalization was further confirmed *in vivo* using a GluA1 Cys811 to serine knock-in mouse (GluA1^C811S^; Itoh et al., 2018). Under basal conditions, these mice show increased phosphorylation levels at Ser831 of GluA1, accompanied by slightly increased expression of GluA1. At the same time, synaptic transmission as well as NMDAR-dependent LTP and LTD remain unaltered. Interestingly, compared to controls, cLTP leads to significantly more spine enlargement in GluA1^C811S^ and GluA1^C811S^ mice are more susceptible to pentylenetetrazole-induced seizures. Confirmation of how relevant AMPAR subunit palmitoylation is for synaptic plasticity *in vivo* comes from two recent studies. Van Dolah et al. (2011) demonstrated that cocaine administration transiently increases palmitoylation of GluA1 and GluA3 in the nucleus accumbens (NAc), a part of the reward system implicated in addictive disorders, leading to the subsequent internalization of AMPAR. Pre-treatment with the palmitoylation inhibitor 2-bromopalmitate (2-BP) before cocaine administration prevents AMPAR internalization and increases the test subjects’ behavioral reaction to cocaine (Van Dolah et al., 2011). Spinelli et al. (2017) showed that feeding mice with high fat diet (HFD) reduced hippocampal LTP and impairs learning and memory in the Morris water maze. In an elegant series of experiments, they found that hippocampal insulin resistance induces overexpression of ZDHHC3 through the transcription factor FoxO3a, which leads to increased palmitoylation of GluA1. Hyperpalmitoylation of GluA1, in turn, reduces its phosphorylation at Ser845, which prevents activity-dependent trafficking to the plasma membrane (Spinelli et al., 2017). Interestingly, the effects of HFD on LTP, learning, and memory are ameliorated by knock-down of ZDHHC3, transfection of double palmitoylation-deficient GluA1, and most importantly, intranasal application of 2-BP (Spinelli et al., 2017).

#### NMDAR

Like AMPAR, NMDAR are tetramers composed of two GluN1 and two GluN2 subunits, with GluN1/2A and GluN1/2B being the predominant isoforms in forebrain although two additional GluN2 genes encode the less prevalent GluN2C and GluN2D subunits (Traynelis et al., 2010; Gray et al., 2011). In contrast to AMPAR, which are permeable for Na^+^ and K^+^, NMDAR also conduct Ca^2+^. GluN2A as well as GluN2B contain clusters of cysteine residues that are palmitoylated (Figure 2C). Cluster I is in the membrane-proximal region of the C-termini (GluN2A-Cys848, Cys853, Cys870; GluN2B-Cys849, Cys854, Cys871) and Cluster II in the more distal C-termini (GluN2A-Cys1214, Cys1217, Cys1236, Cys1239; GluN2B-Cys1215, Cys1218, Cys1239, Cys1242, Cys1245; Hayashi et al., 2009; Figure 2D). Both clusters can be palmitoylated by ZDHHC3, at least when overexpressed (Hayashi et al., 2009). Palmitoylation of GluN2A and GluN2B is activity-dependent; prolonged treatment of cultured cortical neurons with glutamate or bicuculline, which increases activity of glutamatergic synapses, reduces palmitoylation of both subunits. Extended treatment with TTX, which decreases synaptic activity, increases palmitoylation of GluN2A and GluN2B (Hayashi et al., 2005). Palmitoylation of cluster I augments phosphorylation of GluN2A Tyr842 and of GluN2B Tyr1472 by Src family kinases like Fyn, which prevents internalization of the respective receptors (Hayashi et al., 2009). Accordingly, mutating cluster I cysteine residues to serine in either GluN2A or GluN2B reduces synaptic NMDAR currents (Mattison et al., 2012). Palmitoylation of cluster II induces accumulation of NMDAR at the Golgi apparatus, an effect prevented by the introduction of palmitoylation-deficient mutations (Hayashi et al., 2009). It seems therefore likely that depalmitoylation of cluster II is a necessary step allowing externalization of NMDAR (Hayashi et al., 2005). Interestingly, however, while mutation of the cysteines in cluster II increases NMDAR surface expression, it does not increase synaptic currents indicating the existence of additional mechanisms to control NMDAR content in the synapse (Mattison et al., 2012).

#### Synapse Differentiation Induced Gene 1 (SynDIG1)

AMPAR associate with a diverse array of auxiliary proteins influencing their trafficking, localization, and biophysical properties (Jackson and Nicoll, 2011). One established auxiliary AMPAR subunit is the transmembrane protein synapse differentiation induced gene 1 (SynDIG1; Diaz, 2010). In dissociated hippocampal neurons, knockdown or overexpression of SynDIG1 reduces or increases the number, size, and AMPAR content of excitatory synapses, respectively (Kalashnikova et al., 2010). Schaffer collateral LTP is normal in adult but completely absent in 2-week old SynDIG1 knockout animals. In addition, 2-week old knockouts display reduced mEPSPC frequency and amplitude compared to wild-type controls (Chenaux et al., 2016). Intriguingly, blocking neuronal activity of dissociated hippocampal cultures with TTX increases the synaptic localization of SynDIG1 (Kalashnikova et al., 2010). Furthermore, TTX treatment increases density of excitatory synapses from wild-type but not SynDIG1 knockout animals (Chenaux et al., 2016). Recent work demonstrated that SynDIG1 is palmitoylated at two membrane-proximal cysteine residues (Cys191 and Cys192; Kaur et al., 2016). Mutating these sites reduces SynDIG1 protein stability in heterologous cells by preventing transport out of the Golgi apparatus. In neurons, palmitoylation-deficient SynDIG1 stays associated with the Golgi apparatus and is not transported to dendrites. Most interestingly, however, TTX treatment leads to increased SynDIG1 palmitoylation in organotypic hippocampal slice cultures (Kaur et al., 2016). So far, available data do not prove a direct causal relation between SynDIG1 palmitoylation and synaptic localization after TTX treatment. It is, however, tempting to speculate that palmitoylation-supported synaptic localization of SynDIG1 is an additional mechanism reinforcing AMPAR recruitment to the postsynapse during homeostatic plasticity. While the PAT required for SynDIG1 palmitoylation remains undefined, it is known that ZDHHC2 is recruited to the postsynapse after TTX treatment (Noritake et al., 2009). Furthermore, the cysteine residues palmitoylated in SynDIG1 are conserved in other members of the family (SynDIG2–4). It will be interesting to see if these molecules are also palmitoylated, particularly, as one of them, SynDIG4, also influences synaptic plasticity (Matt et al., 2018b).

## Palmitoylation of Cytosolic Molecules

### δ-catenin

δ-catenin modulates *cis*-clustering of N-cadherin (Brigidi and Bamji, 2011). It interacts with PSD-95 (Jones et al., 2002) and GRIP1b (Silverman et al., 2007). A palmitoyl-proteome study identified and confirmed δ-catenin (Kang et al., 2008). The Bamji lab elegantly elucidated the function of δ-catenin palmitoylation in synaptic plasticity using dissociated hippocampal cultures. While δ-catenin is palmitoylated by both ZDHHC5 and ZDHHC20, only ZDHHC5 mediates its activity-induced palmitoylation (Brigidi et al., 2014). Under basal conditions, ZDHHC5 binds to the tyrosine kinase Fyn that is tethered to the postsynaptic membrane by binding to PSD-95. Fyn-mediated phosphorylation of T533 in the endocytic motif prevents ZDHHC5 endocytosis. Increasing neuronal activity by cLTP causes T533 dephosphorylation (presumably by activation of the tyrosine phosphatase STEP61) and endocytosis of ZDHHC5. As a result, DHHC5 localizes to transferrin receptor-positive RE where it is now able to palmitoylate δ-catenin (Brigidi et al., 2015). cLTP induction as well as activity blockade using TTX increase palmitoylation of δ-catenin, which leads to increased co-localization of δ-catenin with N-cadherin at postsynaptic sites, contributing to N-cadherin stabilization at the postsynapse. δ-catenin palmitoylation is required for spine remodeling and AMPAR insertion after cLTP (Brigidi et al., 2014). Knockdown of ZDHHC5 attenuates δ-catenin’s re-localization and N-cadherin stabilization. Palmitoylation of hippocampal δ-catenin and its interaction with N-cadherin are increased 1 h after context-dependent fear conditioning. 24 h after conditioning, δ-catenin palmitoylation levels are back to normal, while its interaction with N-cadherin is still increased (Brigidi et al., 2014). Accordingly, palmitoylation of δ-catenin is temporarily necessary to induce its translocation to the postsynaptic membrane where it supports the strengthening of synaptic connections by stabilizing N-cadherin. δ-catenin palmitoylation, however, is not necessary for maintaining the synapse in a potentiated state, as N-cadherin remains stabilized even after the depalmitoylation of δ-catenin (Brigidi et al., 2014). Most likely, the loss of the δ-catenin palmitate serves to keep the synapse in a plastic state allowing it to undergo further rounds of plastic adaptations, be it additional potentiation or depotentiation. So far, nothing is known about the role of δ-catenin palmitoylation in LTD. Interestingly, ZDHHC5, the PAT responsible for activity-dependent palmitoylation of δ-catenin is also known to palmitoylate GRIP1b, a binding partner of δ-catenin (Silverman et al., 2007; Thomas et al., 2012). This finding invites the speculation that overlap of ZDHHC substrates facilitates the orchestration of postsynaptic responses to plasticity-inducing stimuli.

### Cdc42

Cdc42 is a Rho-related GTPase involved in the organization of the actin cytoskeleton and membrane trafficking. It plays important roles in neurite outgrowth, dendritic arborization and spine formation (Woolfrey and Srivastava, 2016). Knockout of cdc42 in mice prevents LTP, structural LTP (sLTP) of dendritic spines, the postsynaptic sites of glutamatergic synapses, and memory recall in contextual fear conditioning and the Morris Water Maze (Kim et al., 2014). While canonical cdc42 carries a C-terminal prenylation motif, there is a brain-specific splice variant (cdc42-palm) with an alternative C-terminus containing two palmitoylation sites (Cys188, Cys189; Kang et al., 2008; Wirth et al., 2013). Cdc42-palm is necessary for spine formation and synaptogenesis in cultured cortical and hippocampal neurons (Kang et al., 2008; Wirth et al., 2013). Glutamate treatment leads to depalmitoylation of cdc42 and its subsequent removal from dendritic spines (Wirth et al., 2013). Interestingly, cdc42 palmitoylation might be involved in the pathology of 22q11.2 microdeletion syndrome (DiGeorge syndrome), a disease caused by the deletion of 27 known genes including ZDHHC8. 22q11.2 Patients suffer from emotional problems, cognitive deficits, and about 30% of them develop schizophrenia (Mukai et al., 2008). Mice carrying an orthologous deletion are characterized by reduced spine density due to a shortened life span of individual spines (Mukai et al., 2008; Moutin et al., 2017). This spine loss can be rescued by overexpression of either ZDHHC8 or constitutively active cdc42 *in vitro* and *in vivo* (Mukai et al., 2008; Moutin et al., 2017). These studies suggest cdc42 as a relevant target for ZDHHC8 and also provide evidence that impaired palmitoylation of postsynaptic proteins can lead to clinically relevant cognitive neuropsychiatric pathologies.

### LIMK

The actin cytoskeleton in dendritic spines undergoes constant re-arrangement during spine formation and events of synaptic plasticity (Hotulainen and Hoogenraad, 2010; Bosch et al., 2014). A pivotal supporter of F-actin polymerization is LIM kinase-1 (LIMK1) which phosphorylates and inactivates the F-actin-severing protein cofilin (Hotulainen and Hoogenraad, 2010). LIMK1 contains conserved N-terminal palmitoylation sites at Cys7 and Cys8 (George et al., 2015). Palmitoylation-deficient LIMK1 is not targeted to dendritic spines and fluorescence recovery after photobleaching (FRAP) of green fluorescent protein (GFP)-tagged actin is strongly reduced after acute knockdown of LIMK1 or replacement with palmitoylation-deficient LIMK1 (George et al., 2015), indicating reduced turnover of F-actin. Additionally, sLTP, as assessed by measuring the growth of an individual spine after glutamate stimulation by optical uncaging, is also reduced after acute knockdown of LIMK1 or replacement with palmitoylation-deficient LIMK1 (George et al., 2015; Montersino and Thomas, 2015). Most interestingly, activation of LIMK1 through phosphorylation by its upstream activator p21-activated kinase (PAK) is only possible if LIMK1 is palmitoylated at both Cys7 and Cys8 (George et al., 2015).

### Activity-Regulated Cytoskeleton-Associated Protein (Arc)/Arg3.1

Activity-regulated cytoskeleton-associated protein (Arc), also known as activity-regulated gene of 3.1 kb (Arg3.1) is one of the earliest genes known to be induced by synaptic activity with transcripts appearing as early as 5 min after stimulation (Ramírez-Amaya et al., 2005). Arc induction was demonstrated after a variety of neuronal stimulation paradigms like seizures (Link et al., 1995; Lyford et al., 1995) and learning experience (Montag-Sallaz and Montag, 2003; Korb and Finkbeiner, 2011) as well as LTP (Waltereit et al., 2001), LTD (Waung et al., 2008), and synaptic scaling (Korb et al., 2013). After induction, Arc mRNA is rapidly transported to the postsynapse where it is locally translated and rapidly degraded (Bramham et al., 2010). Among many other functions in synaptic plasticity, Arc promotes AMPAR endocytosis during LTD by interacting with clathrin-mediated endocytosis (Chowdhury et al., 2006; Rial Verde et al., 2006) and stabilizes LTP through its interaction with the F-actin cytoskeleton (Messaoudi et al., 2007). Arc undergoes palmitoylation at three cysteine residues in the N-terminal half of the so far structurally uncharacterized protein (Barylko et al., 2018). Transfection of neurons in organotypic slice cultures with the transcription factor MEF2 leads to synaptic depression and synapse elimination. This MEF2-dependent paradigm of synaptic plasticity was established in earlier work of the same laboratory (Wilkerson et al., 2014). Co-transfection of MEF2 with wild-type but not palmitoylation-deficient Arc in slices from Arc knockout mice leads to synaptic depression (Barylko et al., 2018). This finding shows that palmitoylation of Arc is necessary for MEF2-dependent synaptic depression. However, more work is required here, particularly in the light of the still incompletely understood cell biology of Arc. It would also be interesting to see, which ZDHHC is responsible for the palmitoylation of Arc.

## Palmitoylation of Scaffold Proteins

Synaptic plasticity depends on the spatiotemporally regulated function of many different signaling molecules. For LTP, among other molecules, a number of protein kinases like cAMP-dependent protein kinase (PKA), protein kinase C (PKC), and Ca^2+^-calmodulin-dependent kinase II (CaMKII) are important for the underlying increase in AMPAR open probability, conductance, and postsynaptic localization (Malenka and Bear, 2004; Sanderson and Dell’Acqua, 2011; Lüscher and Malenka, 2012; Hell, 2014; Woolfrey and Dell’Acqua, 2015). For LTD, the same is true for an array of protein phosphatases such as protein phosphatases 1 (PP1), 2A (PP2A) and 2B (PP2B, calcineurin CaN), which contribute to the reduction in synaptic strength (Malenka and Bear, 2004; Sanderson and Dell’Acqua, 2011; Lüscher and Malenka, 2012; Woolfrey and Dell’Acqua, 2015). In both cases, it is necessary that these and other molecules are localized with high spatial and temporal fidelity into multiprotein signaling complexes by an array of scaffolding, anchoring, and adaptor proteins. Among others, these include PSD-95, which is essential for the postsynaptic targeting of AMPAR (Won et al., 2017), the A-kinase anchoring protein (AKAP) family, which links some of the kinases and phosphatases to their postsynaptic targets (Sanderson and Dell’Acqua, 2011), and the inhibitory scaffold protein gephyrin, which helps anchoring GABA_A_R and glycine receptors at inhibitory synapses (Tyagarajan and Fritschy, 2014).

### AKAP5

**Figure 3:**
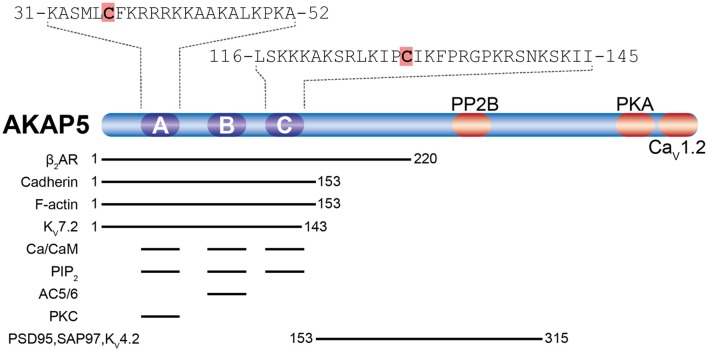
Palmitoylation of A-kinase anchoring protein 5 (AKAP5). Shown are AKAP5 palmitoylation sites (orange shading) within the N-terminal polybasic regions A and C in relation to known binding sites for AKAP5 associated proteins (reviewed in Sanderson and Dell’Acqua, 2011; Woolfrey and Dell’Acqua, 2015; Patriarchi et al., 2018). Residue numbering refers to human AKAP5. The β_2_-adrenoreptor (β_2_AR), cadherin, F-actin, and the voltage-activated potassium channel K_V_7.2 interact with the N-terminal half of AKAP5. All three polybasic regions bind to Ca^2+^/Calmodulin (Ca^2+^/CaM) and phosphatidylinositol 4,5-bisphosphate (PIP_2_). Adenylyl cyclases 5 and 6 (AC5/6) bind polybasic region B and PKC binds to polybasic region A. postsynaptic density-95 (PSD-95) and SAP97 interact through their Src homology 3 (SH3) and GK domains with the center of AKAP5, which also binds the K^+^ channel K_V_4.2. PP2B interacts near the center of AKAP5 and protein kinase A (PKA) with a motif about 20 residues upstream of the C-terminus, while the α_1_1.2 subunit of Ca_V_1.2 binds to the last ~15 residues at the C-terminus.

The about 50 members of the AKAP family are defined by the presence of a short amphipathic α-helix, which binds the regulatory subunits of the PKA holoenzyme with high affinity (Carnegie et al., 2009; Sanderson and Dell’Acqua, 2011). Of particular interest is AKAP5 (human AKAP79/rodent AKAP150), a postsynaptic scaffolding protein that integrates an array of plasticity-related ion channels, neurotransmitter receptors, scaffolding, and signaling proteins. These include PKA, PKC, several adenylate cyclase (AC) isoforms, the phosphatase CaN, the scaffolding proteins PSD-95 and SAP97, as well as the L-type Ca^2+^ channel Ca_V_1.2 (see Sanderson and Dell’Acqua, 2011; Woolfrey and Dell’Acqua, 2015; Patriarchi et al., 2018; Figure 3). AKAP5 was identified as a palmitoylated postsynaptic protein in a proteomic study (Kang et al., 2008). Palmitoylation happens at Cys36 and Cys129, both located in the N-terminal polybasic regions (Figure 3), which are necessary to target AKAP5 to phosphatidylinositol-4,5-bisphosphate (PIP2) containing membranes like the plasma membrane or REs (Dell’acqua et al., 1998; Delint-Ramirez et al., 2011; Woolfrey et al., 2015). cLTD causes depalmitoylation and removal of AKAP5 from spines *in vitro*, while kainate-induced seizure activity *in vivo* leads to increased palmitoylation and association with synaptic lipid rafts (Keith et al., 2012). cLTP induction activates ZDHHC2 located in RE, which leads to AKAP5 palmitoylation and re-location to RE (Woolfrey et al., 2015). RE-localized AKAP5 forms complexes with PKA, CaN, and SAP97, which are subsequently delivered to the postsynapse by exocytosis (Keith et al., 2012). If Cys36 and Cys129 of AKAP5 are mutated to preclude its palmitoylation, cLTP induction does not lead to increased RE trafficking, spine enlargement, and AMPAR recruitment to the postsynapse (Keith et al., 2012; Woolfrey et al., 2015). Similar effects are observed after knock down of ZDHHC2. Here cLTP does not increase exocytosis of SEP-tagged transferrin receptors in contrast to control conditions, underlining the importance of AKAP5 palmitoylation for RE exocytosis (Woolfrey et al., 2015). Under basal conditions, knock down of ZDHHC2 for 48 h leads to increased mEPSC frequency and amplitude in cultured hippocampal neurons, together with increased PSD-95 cluster density. cLTP induction after knock down of ZDHHC2, however, leads to a massive decrease of mEPSC frequency and amplitude within 30 min (Woolfrey et al., 2015). This is in accordance with a model of reduced postsynaptic stability after Ca^2+^ influx (e.g., after cLTP) in which postsynaptic components are more labile. Apparently, palmitoylation of ZDHHC2 targets such as AKAP5 and PSD-95 confers some stability to the postsynaptic assembly that increases synaptic strength under basal conditions, but when lacking, promotes disassembly of the synapse after stimulation. In contrast to PSD-95, palmitate cycling does not seem to be important for AKAP5 function, as a palmitoylation-deficient myristoylated protein behaves like the wild-type in terms of RE and PSD recruitment and also supports cLTP like wild-type (Woolfrey et al., 2015). Knock-in mice carrying palmitoylation deficient AKAP5 show increased numbers of postsynaptic GluA1-homotetrameric Ca^2+^-permeable AMPAR (CP-AMPAR). In these mice, it is not possible to observe CP-AMPAR-dependent LTP, while LTD and GluA2-dependent LTP remain unchanged (Purkey et al., 2018).

### GRIP1b

Both GRIP1 and its close homolog GRIP2 are postsynaptic scaffolding proteins containing seven PDZ-type protein interaction domains of which PDZ5 directly binds GluA2 (Kim and Sheng, 2004). For each, GRIP1 and GRIP2, one of two N-terminal splice variants, GRIP1b (Yamazaki et al., 2001) and GRIP2b (also known as palmitoylated AMPAR binding protein long-form, pABP-L; Desouza et al., 2002; Misra et al., 2010) were shown to be palmitoylated. The N-terminal 45 residues of the non-palmitoylated GRIP1a are replaced by 19 alternative residues in GRIP1b (Yamazaki et al., 2001), while GRIP2b differs from GRIP2a only by an alternative sequence of the first 18 N-terminal residues (Desouza et al., 2002). Activity-dependent palmitoylation was reported for GRIP1b (Hanley and Henley, 2010; Thomas et al., 2012), but not GRIP2b. ZDHHC5 and ZDHHC8 feature C-terminal interaction sites for PDZ domains and directly interact with GRIP1b (Thomas et al., 2012). This PDZ interaction is necessary for the palmitoylation of GRIP1b in heterologous cells and cultured neurons, where palmitoylated GRIP1b localizes to endosomal compartments (Hanley and Henley, 2010; Thomas et al., 2012). NMDA treatment of cultured neurons leads to temporary removal of SEP-tagged GluA2 from the cell surface. Co-transfection of ZDHHC5 accelerates the recovery of SEP-GluA2 from endocytosis (Thomas et al., 2012). In contrast, using a surface biotinylation assay, Hanley and Henley (2010) observe a stronger reduction of surface GluA2 after treating hippocampal cultures with NMDA when they co-transfect GRIP2b. This discrepancy might be explained by the fact that Hanley and Henley (2010) harvest their cells 13 min after the onset of NMDA stimulation, which is approximately the point in time at which Thomas et al. (2012) observe the maximum number of endocytosed channels before recovery. Furthermore, another study did not find any differences in GRIP1b palmitoylation after kynurenic acid stimulation of hippocampal neurons (Noritake et al., 2009). The role of activity-dependent GRIP1b palmitoylation in synaptic plasticity is thus incompletely understood.

### PICK1

PICK1 is a PDZ- and BAR-domain containing protein, which interacts with a variety of other proteins including GluA2, GRIP, PKCα, cdc42 (Kim and Sheng, 2004; Li et al., 2016) and also ZDHHC8, which binds *via* its C-terminal PDZ ligand (Thomas et al., 2013). PICK1 is necessary for LTD induced by glutamate application in cultured cerebellar Purkinje cells (PCs). LTD is abolished by incubation with 2-BP. In PC from PICK1 knock-out mice, LTD is rescued in cells transfected with wild-type but not palmitoylation deficient PICK1. LTD in wild-type PC is also prevented by knockdown of ZDHHC8, which can be rescued by co-transfection of prenylated PICK1 (Thomas et al., 2013). The report by Thomas et al. (2013) constituted the first evidence that altered palmitoylation of one specific protein leads to altered synaptic plasticity. Earlier reports using 2-BP (e.g., Van Dolah et al., 2011) could only demonstrate an association between palmitoylation and changes in plasticity So far, there are no reports concerning the role of PICK1 palmitoylation in hippocampal neurons. Interestingly, however, PICK1 interacts with GRIP1 and both proteins are palmitoylated by ZDHHC8. This is again a hint towards certain ZDHHC being “master regulators” of certain clusters of plasticity-related effectors.

### Gephyrin

Like in excitatory synapses, specific scaffold proteins organize the structure and composition of inhibitory synapses. The most extensively studied inhibitory scaffold is gephyrin, which is essential for postsynaptic clustering glycine receptors and GABA_A_ receptors as well as for the plasticity of inhibitory synapses (Tyagarajan and Fritschy, 2014). A proteomic study identified gephyrin as potentially palmitoylated protein (Kang et al., 2008). Gephyrin interacts with ZDHHC12, which palmitoylates Cys212 and Cys284 in gephyrin (Dejanovic et al., 2014). Co-expression of ZDHHC12 with gephyrin in HEK293 cells enhances gephyrin palmitoylation. In neurons, ZDHHC12 overexpression increases the size of gephyrin clusters and the amplitude of miniature inhibitory postsynaptic potentials (mIPSC). It seems most likely that palmitoylation of gephyrin happens at the Golgi apparatus membrane, where a strong co-localization with ZDHHC12 is found (Dejanovic et al., 2014). Ectopic expression of palmitoylation-deficient gephyrin leads to an increased number of gephyrin clusters on the dendritic shaft, which unlike the wild-type, do not overlap with GABA_A_ receptor clusters (Dejanovic et al., 2014). Most interestingly, however, is the fact that palmitoylation levels of gephyrin are regulated by the activity of GABAergic synapses. Bath application of the receptor agonist GABA increases gephyrin palmitoylation, while it is decreased after application of the selective GABA_A_ receptor antagonist bicuculline (Dejanovic et al., 2014). This work demonstrates that activation of specific PATs not only influence the plasticity of excitatory but also of inhibitory synapses.

## The Role of PSD-95 Palmitoylation in Synaptic Plasticity

**Figure 4:**
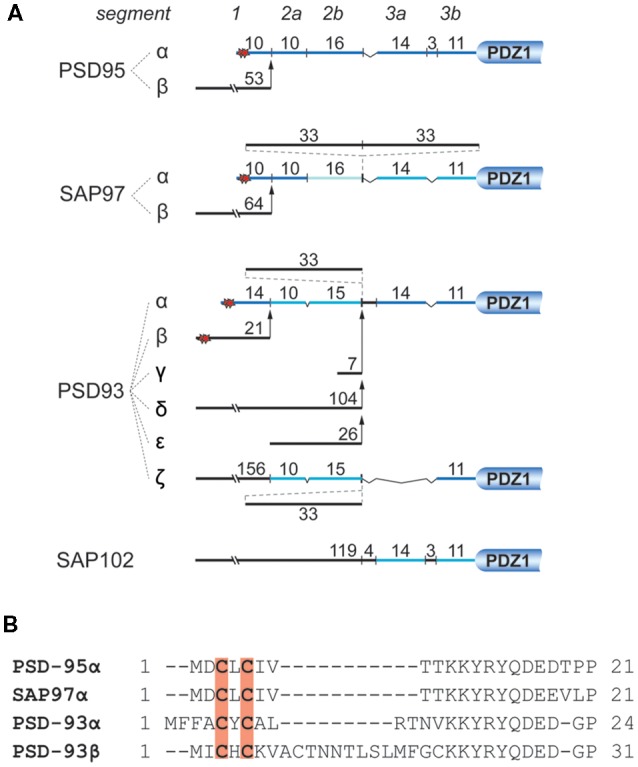
N-terminal splice variants of PSD-95, PSD-93, SAP97, and SAP102. **(A)** Depicted are segments classified by sequence homology with their number of residues. Palmitoylation sites are indicated by red stars. For instance PSD-95 and SAP97 exist in two N-terminal splice variants, an α isoform, which is palmitoylated within its first 10 residues (Cys3 and Cys5), and a β variant containing an L27 interaction motif encoded on alternatively spliced exons (Chetkovich et al., 2002; Schlüter et al., 2006). PSD-93 has six N-terminal splice variants, two of which are palmitoylated: PSD-93α, which is most similar to PSD-95α, and PSD93β (Parker et al., 2004; Krüger et al., 2013). The N-terminus of SAP102 only exists in one splice variant which contains a L27 domain and is not palmitoylated (Müller et al., 1996). **(B)** Sequence alignment of the N-termini of PSD-95α, SAP97α, PSD-93α, and PSD-93β. Red shading indicates palmitoylated cysteines corresponding to red stars in **(A)**.

With roughly 300 molecules per postsynaptic site, PSD-95 is one of the most abundant scaffold proteins at excitatory synapses (Cheng et al., 2006; Sheng and Kim, 2011). PSD-95 directly or indirectly interacts with cell-adhesion molecules, neurotransmitter receptors and cytoskeletal proteins to regulate the structural organization of the PSD, synaptic transmission (Béïque et al., 2006; Chen et al., 2011; Hafner et al., 2015) and synaptic plasticity (Migaud et al., 1998; Stein et al., 2003; Ehrlich and Malinow, 2004; Béïque et al., 2006; Elias et al., 2006; Ehrlich et al., 2007). Therefore, proper synaptic localization of PSD-95 is important for neuronal development and function. So far, a number of mechanisms have been reported to regulate the postsynaptic localization of PSD-95 including interaction with other proteins (Hruska et al., 2015; Yadav et al., 2017; Chowdhury et al., 2018; Matt et al., 2018a) and posttranslational modification such as phosphorylation (Morabito et al., 2004; Kim et al., 2007; Steiner et al., 2008; Nelson et al., 2013), palmitoylation (Craven et al., 1999; El-Husseini et al., 2000a), ubiquitination (Waataja et al., 2008), S-nitrosylation (Ho et al., 2011) and neddylation (Vogl et al., 2015). Among these, palmitoylation at Cys3 and Cys5 of PSD-95 is considered a central mechanism by which PSD-95 is anchored at synaptic sites, where it regulates synaptic strength through modulating the availability of AMPAR. The importance of palmitoylation for postsynaptic localization of PSD-95 was illustrated using PSD-95 tagged with photoactivatable GFP. Within 30 min, turnover of wild-type PSD-95 within a single spine is about 10%, but almost 100% for palmitoylation-deficient PSD-95 (Sturgill et al., 2009). Of the other highly homologous members of the PSD-95 family, SAP97 and PSD-93 exist in N-terminal splice variants containing palmitoylation sites very similar to PSD-95 (Figure 4).

Isoform-specific localization of SAP97 within a spine and concomitant regulation of synaptic strength and plasticity have been reported. Biochemistry and electron microscopy using overexpressed SAP97 indicate that palmitoylated SAP97α is associated with the PSD, whereas SAP97β containing L27 domain instead of palmitoylation motif at its N-terminus shows primarily non-PSD distribution within spines (Waites et al., 2009). Accordingly, it is believed that SAP97α and SAP97β can differentially regulate synaptic strength and plasticity by regulating the availability of synaptic and extrasynaptic glutamate receptors, respectively (Schlüter et al., 2006; Waites et al., 2009; Li et al., 2011). There is also evidence that PSD-95 exists in analogous α and β isoforms with PSD-95α containing the classic, palmitoylated N-terminus and PSD-95β an L27 domain at its N-terminus (Schlüter et al., 2006). About 90% of brain PSD-95 is the α-isoform (Chetkovich et al., 2002), whereas the prevalence of SAP97β, which is the main SAP97, is estimated to be ~16% of PSD-95α in the hippocampus (Schlüter et al., 2006), the role of SAP97α, which is even less abundant than SAP97β is expected to be limited. Very little is known about the effects of PSD-93 palmitoylation. Two of its N-terminal splice variants are palmitoylated (Figure 4; El-Husseini et al., 2000b). PSD-93 palmitoylation, however, might not be necessary for synaptic targeting of PSD-93 (Firestein et al., 2000). Still, overexpression of either PSD-93α and PSD-93β seems to compensate the loss of postsynaptic AMPAR after knock-down of PSD-95 (Krüger et al., 2013).

### PSD-95 Palmitoylation

Palmitoylation of PSD-95 at Cys3 and Cys5 is necessary for its interaction with the K^+^ channel K_V_1.4 (Topinka and Bredt, 1998) and for postsynaptic clustering of PSD-95 (Craven et al., 1999). Other lipid modifications such as myristoylation or geranylation cannot functionally substitute for palmitoylation of PSD-95. Prenylated PSD-95 can form clusters in COS7 cells and neurons, however, compared to wild-type PSD-95, those tend to be bigger and mis-localized (El-Husseini and Bredt, 2002). This is indicative of a specific role of palmitoylation in postsynaptic targeting (Craven et al., 1999), possibly by localized palmitoylation by spine- or PSD-targeted PATs as proposed above, which could augment localized attachment of the various palmitoylated proteins to the plasma membrane of spines. To identify PSD-95 palmitoylating enzyme(s), 23 mammalian ZDHHC proteins were isolated based on their sequence homology with the DHHC region of GODZ (Golgi apparatus-specific protein with the DHHC zinc finger domain, ZDHHC3; Fukata et al., 2004). Two subfamilies of ZDHHC proteins, ZDHHC3/7 and ZDHHC2/15, palmitoylated PSD-95 *in vitro* and in heterologous cells (Fukata et al., 2004). ZDHHC15 and 7 are minor isoform in the hippocampus. Subsequent work focused on ZDHHC2 and 3, which turned out to be differentially distributed in neurons with ZDHHC2 being present in many subcellular fractions including PSD while ZDHHC3 being present only in the fraction containing light membranes (typically called P3). Consistent with this finding, ZDHHC2 was localized in cell bodies and dendrites whereas ZDHHC3 signal was detected specifically at the Golgi apparatus in the immunostaining of dissociated hippocampal neurons (Noritake et al., 2009). Also, ZDHHC2 was later found to be present in REs and neuronal surface including spines when ZDHHC3 was not detected on the cell surface (Fukata et al., 2013). Remarkably, only dendritically localized ZDHHC2, but not Golgi apparatus-resident ZDHHC3, mediates activity-regulated dynamic palmitoylation of PSD-95. When synaptic activity was blocked by TTX or kynurenic acid, PSD-95 was rapidly palmitoylated and formed clusters at the synapse; ZDHHC2 translocated to the PSD to mediate this effect. Therefore, it seems that ZDHHC3 palmitoylates various newly synthesized proteins including PSD-95 at the Golgi apparatus in the soma. ZDHHC2 on the other hand is responsible for more dynamic and local palmitoylation of PSD-95 that is regulated by synaptic activity. This suggests that individual ZDHHC proteins are differentially distributed and regulated for compartmentalized palmitoylation of PSD-95 in response to synaptic activity.

PSD-95 exists in nanodomains within a spine and undergoes continuous palmitoylation/depalmitoylation cycles driven by local palmitoylating activity (Fukata et al., 2013). Furthermore, these reactions occur in a single spine without supply of new PSD-95 from outside the spine, suggesting that spines contain both palmitoylating and depalmitoylating enzymes. Importantly, activity-dependent insertion of ZDHHC2 into the plasma membrane is responsible for rapid organization of PSD-95 nanodomains through palmitoylation; when ZDHHC2 was trapped in ER by the addition of an ER-retention motif at its C-terminus, the number of nanodomains containing palmitoylated PSD-95 was dramatically reduced. These findings strongly suggest that local, dynamic palmitoylation of PSD-95 within a spine by ZDHHC2 not only maintains PSD-95 nanodomains by preventing lateral diffusion or endocytosis of membrane-bound PSD-95, but also drives reorganization of PSD structure in a synaptic activity-dependent manner. Therefore, local palmitoylation of a key scaffolding protein in the PSD allows synapses to respond structurally and functionally (which will be described later) in a neuronal activity-dependent manner.

### The Effect of PSD-95 Palmitoylation on Synaptic Plasticity and Homeostatic Scaling

PSD-95 overexpression increases the amplitude of the excitatory postsynaptic current (EPSC; Schnell et al., 2002; Béïque and Andrade, 2003; Ehrlich and Malinow, 2004; Elias et al., 2008) whereas reducing PSD-95 levels results in the opposite effect (Elias et al., 2006; Schlüter et al., 2006; Ehrlich et al., 2007). Consistent with these results, PSD-95 overexpression occludes LTP and enhances LTD (Stein et al., 2003; Ehrlich and Malinow, 2004), and knockout or knockdown of PSD-95 reduces AMPAR currents and enhances LTP (Béïque et al., 2006; Elias et al., 2006; Ehrlich et al., 2007). Because palmitoylation is a major determinant of synaptic PSD-95 clustering, it was anticipated that its palmitoylation is closely related to changes in synaptic strength. Consistent with this notion, activation of glutamate receptors can disperse synaptic clusters of PSD-95 through depalmitoylation and consequently lead to the selective loss of postsynaptic AMPAR without affecting NMDAR (El-Husseini and Bredt, 2002). Furthermore, inhibition of palmitoylation with 2-BP in hippocampal neurons reduces the amplitude and frequency of AMPAR mediated mEPSC (El-Husseini and Bredt, 2002; Schnell et al., 2002). The PSD-95 interaction with the auxiliary TARP subunits stargazin (γ2) and γ8 anchors AMPAR at postsynaptic sites, which requires palmitoylation of PSD-95 (Chen et al., 2000; Schnell et al., 2002). Palmitoylation-deficient C3S/C5S mutant PSD-95 failed to enhance AMPAR EPSCs (Schnell et al., 2002; Ehrlich and Malinow, 2004).

Homeostatic synaptic scaling refers to the ability of neurons to proportionally scale the strength of all of their synapses down or up to remain within an appropriate range in response to prolonged elevation or reduction in neuronal network activity, respectively. Postsynaptic accumulation of PSD-95 is bidirectionally modulated by such chronic activity changes (Kim et al., 2007) and PSD-95 is necessary for both scaling up and down (Sun and Turrigiano, 2011). Chronic blockade of synaptic activity by TTX increased PSD-95 palmitoylation and resulted in the accumulation of PSD-95 at synaptic sites possibly due to stimulation of DHHC2 translocation by TTX treatment (Noritake et al., 2009; Fukata et al., 2013) whereas prolonged elevation of synaptic activity by bicuculline reduced PSD-95 palmitoylation (Chowdhury et al., 2018). In parallel, AMPAR are recruited to or dispersed from synapses, respectively, suggesting that a dynamic palmitoylation cycle of PSD-95 within a spine modulates bidirectional homeostatic AMPAR plasticity.

**Figure 5:**
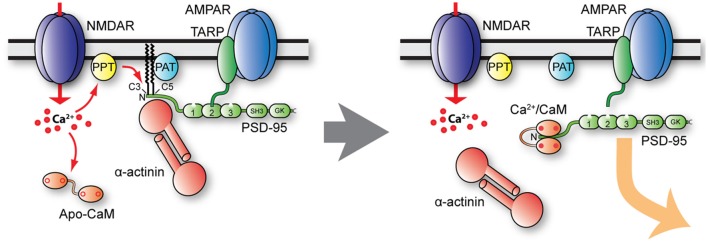
Model of postsynaptic PSD-95 anchoring and its displacement upon Ca^2+^ influx. Under basal conditions (left), PSD-95 is kept at postsynaptic sites by palmitoylation (PAT protein ZDHHC8) and binding to α-actinin. Ca^2+^ influx likely stimulates PSD-95 depalmitoylation (PPT), which allows binding of Ca^2+^/CaM to shift the equilibrium of palmitoylated, α-actinin—bound PSD-95 to non-palmitoylated PSD-95 in part by Ca^2+^/CaM capping of the N-terminus of PSD-95, thereby preventing re-palmitoylation. Ca^2+^/CaM also competes with and thereby displaces α-actinin from the N-terminus of PSD-95 when it is depalmitoylated.

Palmitoylation of PSD-95 on Cys3 and Cys5 is regulated in various ways. Cys3 and Cys5 are S-nitrosylated by NO when Ca^2+^ influx through NMDAR activates nNOS *via* calmodulin (Ho et al., 2011). This nitrosylation reduces both PSD-95 palmitoylation and the number of synaptic PSD-95 clusters in cerebellar granule cell. Similarly, PSD-95 nitrosylation is increased after inhibition of palmitoylation with 2-BP and in ZDHHC8 knock-out mice (Ho et al., 2011). Furthermore, Ca^2+^ influx through NMDAR triggers binding of Ca^2+^-calmodulin to the N-terminus of PSD-95 (amino acid 1–13) to antagonize palmitoylation (Zhang et al., 2014). Consequently, calmodulin-bound and depalmitoylated PSD-95 leaves the postsynaptic site (Figure 5). Furthermore, α-actinin was recently found to bind to the N-terminus of PSD-95, thereby anchoring PSD-95 at postsynaptic sites without affecting its palmitoylation (Matt et al., 2018a). The α-actinin binding region overlaps with the calmodulin binding region and Ca^2+^-calmodulin displaces α-actinin from PSD-95 upon NMDAR-mediated Ca^2+^ influx (Matt et al., 2018a). Accordingly, upon Ca^2+^ influx, Ca^2+^-calmodulin binds to N-terminus of PSD-95 to displace α-actinin and prevent re-palmitoylation. As a result, PSD-95 is released from spines (Figure 5). Ubiquitination of PSD-95 at multiple lysine residues contributes to the removal of PSD-95 from the synapse and AMPAR internalization in NMDAR-dependent LTD (Colledge et al., 2003). Thus, various mechanisms are in place to govern synaptic strength by regulating postsynaptic PSD-95 and thereby AMPAR content.

### Other Aspects of PSD-95 Palmitoylation

In addition to simply localizing PSD-95 at the synapse and thus providing AMPAR anchoring “slots” at the PSD, PSD-95 palmitoylation may contribute to the regulation of synaptic strength by (re)organization of the entire PSD structure. Palmitoylation changes PSD-95 from a compact conformation as presumably prevalent outside synapses to an extended one perpendicular to the PSD membrane, with its palmitoylated N-terminal domain at the membrane (Chen et al., 2011; Jeyifous et al., 2016). Importantly, PSD-95 associates with AMPAR (*via* TARPs) and NMDAR (*via* GluN2B) only when palmitoylated and in its extended configuration. SAP97 also showed extended and compact conformation within and outside of the PSD, respectively. SAP97 configuration, however, is regulated by interactions with its N-terminal L27 domain. SAP97 becomes extended when CASK, one of its binding partners, associates with its L27 domain or SAP97 forms a homodimer through its L27 domain (Lin et al., 2013). In addition, unlike PSD-95, SAP97 interacts with the AMPAR GluA1 subunit in its compact conformation and with the NMDAR GluN2B subunit in its extended conformation (Lin et al., 2013), and orients parallel to the PSD membrane (Jeyifous et al., 2016). These differences between PSD-95 and SAP97 in glutamate receptor binding and orientation at postsynaptic sites seem to contribute to separate AMPAR and NMDAR nanodomains in the PSD (Chen et al., 2008, 2011). In the NMDA receptor nanodomain, NMDAR are scaffolded by both PSD-95 and SAP97, creating a dense lattice structure, which might prevent access of palmitoylation/depalmitoylation enzymes. The more dynamic AMPAR nanodomain is formed by weaker interaction between PSD-95 and TARPs, allowing easier access of palmitoylating/depalmitoylating enzymes. As a result, dynamic palmitoylation cycling changes PSD-95 conformation and TARP binding, thereby regulating the number of AMPAR slots in AMPAR nanodomains. This hypothesis is consistent with the observation that changing PSD-95 palmitoylation in PSDs altered PSD-95 and AMPAR but not NMDAR levels (Jeyifous et al., 2016), and that AMPAR nanodomains were lost while NMDAR nanodomains were preserved upon PSD-95 knockdown (Chen et al., 2011). Adopting an extended conformation likely also contributes to binding of PSD-95 to stargazin and potentially other TARPs, whose C-termini also undergo an extension away from the plasma membrane upon their phosphorylation by CaMKII (Hafner et al., 2015), which is an important molecular component of synaptic plasticity (Hell, 2014; Patriarchi et al., 2018).

## Perspective

Postsynaptic proteins are more likely to be palmitoylated than most other proteins; at the same time, mutations of palmitoylation sites are associated with a number of diseases and disorders of the nervous system (Sanders et al., 2015; Cho and Park, 2016; Zaręba-Kozioł et al., 2018). Therefore, it is safe to assume that palmitoylation is an important factor in the proper development and function of synapses.

While the significance of protein palmitoylation in neural development was exhaustively discussed elsewhere (El-Husseini et al., 2002; Fukata and Fukata, 2010; Blaskovic et al., 2014; Globa and Bamji, 2017), it seems relevant to mention an emerging aspect of palmitoylation of PSD-95 and other postsynaptic proteins, the recently introduced concept of phase transition (Zeng et al., 2016, 2018). When PSD-95 and other purified postsynaptic protein components like SynGAP, GKAP/SAPAP, Shank3, and Homer3 are mixed in appropriate stoichiometry *in vitro*, they spontaneously form highly concentrated phase-separated liquid-like droplets reminiscent of the PSD (Zeng et al., 2016, 2018). These droplets absorb soluble fragments of the C-terminus of the NMDAR GluN2B subunit, which binds with its C-terminus to PDZ1 and PDZ2 domains of PSD-95 (Kornau et al., 1995; Irie et al., 1997; Kim et al., 1997), while they repel the inhibitory scaffold protein gephyrin. The same protein mixture induces clustering of the GluN2B C-terminus when attached to an artificial lipid bilayer and promotes actin bundle formation. Remarkably, upon addition of Homer1a, which antagonizes spine morphogenesis, synaptic targeting of PSD scaffolding proteins, AMPAR surface expression and synaptic transmission (Sala et al., 2003), the PSD condensates gradually disperse (Zeng et al., 2018). It is conceivable that synapse formation *in vivo* follows similar principles of self-organization. Considering the high abundance of PSD-95 and how essential it is for this phase transition, it is conceivable that PSD-95, when immobilized at the PSD by palmitoylation, acts as a seed molecule to initiate phase transition. We propose that binding of PSD-95 to α-actinin, which is concentrated in spines due to its binding to F-actin, mediates the initial recruitment of PSD-95 to spines, supported by localized palmitoylation. When reaching the critical concentration, the forces that drive phase separation would foster its clustering into nanodomains. This mechanism could be augmented by PSD-95 adopting its extended conformation as driven by palmitoylation, which in turn would provide maximum capacity for protein interactions engaging its PDZ, SH3, and GK domains. Effects of phase transition might be complemented by the concentration of raft-promoting lipids in the plasma membrane of postsynaptic termini that occurs during synaptogenesis in parallel to the appearance of palmitoylated PSD-95 in the PSD (Tulodziecka et al., 2016). It is quite possible that palmitoylation of postsynaptic proteins like PSD-95 and the development of postsynaptic membranes high in raft-promoting lipids might be coordinated by the preferred interaction of membrane lipids with the palmitate anchors of immobilized postsynaptic proteins (Tulodziecka et al., 2016). Palmitoylation of postsynaptic proteins might thus represent another parameter in the self-organized assembly of the PSD.

In addition to affecting synaptic development, the disproportionally high number of palmitoylated proteins at postsynaptic sites also influences synaptic function, of which synaptic plasticity is but one aspect. In this review article, we have discussed a number of postsynaptic proteins, whose palmitoylation affects synaptic plasticity, usually by promoting postsynaptic localization. Although it is still not completely clear whether palmitoylation by itself can mediate postsynaptic localization of these proteins, it appears likely that it mostly enhances membrane association, while postsynaptic localization is ensured by protein-protein interactions. The preferred association of palmitoylated PSD-95 with membranes rich in rafts-promoting lipids (Tulodziecka et al., 2016) suggests that palmitoylated proteins might be more likely to associate with raft-like membrane areas like those found at postsynaptic sites. The fact that other lipid modifications like prenylation (Thomas et al., 2013) and myristoylation (Woolfrey et al., 2015) are sufficient to rescue certain palmitoylation-dependent functions of PICK1 and AKAP5, respectively, suggests that lipid modification and the resulting membrane association contribute in a non-specific manner to postsynaptic clustering. In other words, these modifications help recruit proteins to postsynaptic sites by mediating membrane interactions rather than *via* specific anchoring mechanisms. So far, however, there is no indication that palmitoylation is sufficient for postsynaptic targeting.

Another question is how palmitoylation differs from other lipid modifications like prenylation or myristoylation (Nadolski and Linder, 2007; De and Sadhukhan, 2018). Notably, of all lipid modifications only palmitoylation is reversible, making it suitable for regulating synaptic development and function, both of which are characterized by plastic changes. Interestingly, this reversibility is not necessary for the function of AKAP5, as its palmitoylation-deficient myristoylated form supports plasticity like the wild-type (Woolfrey et al., 2015). At the same time, cdc42 exists in different splice variants, a canonical form which is prenylated, and a neuronal form, which is palmitoylated (Kang et al., 2008; Wirth et al., 2013). In this case, the reversibility of the lipid modification seems to be important for cdc42’s function in synaptic development and function (Hayashi et al., 2005; Kang et al., 2008; Mattison et al., 2012; Wirth et al., 2013).

Palmitoylation of GluA1 was shown to reduce its phosphorylation which in turn increases AMPAR stability in the postsynaptic membrane (Hayashi et al., 2005; Lin et al., 2009), while LIMK activation through phosphorylation by PAK is only possible if LIMK is palmitoylated (George et al., 2015). It would be interesting to see, if a similar interplay between phosphorylation and palmitoylation could also be observed in other postsynaptic proteins. It is conceivable, that the palmitoylation state of PSD-95 at Cys3 and Cys5 influences its phosphorylation at Thr19, a phosphorylation site known to modulate the mobilization of PSD-95 (Nelson et al., 2013).

Further defining how palmitoylation affects postsynaptic structure and function undoubtedly will provide important insight into the molecular mechanisms that govern synapse formation and synaptic plasticity.

## Author Contributions

All authors contributed to the writing of this manuscript. LM, KK, and DC wrote the first draft. LM, KK, DC and JH edited the draft. LM prepared the figures. LM and JH finalized the manuscript.

## Conflict of Interest Statement

The authors declare that the research was conducted in the absence of any commercial or financial relationships that could be construed as a potential conflict of interest.
